# From Micro- to Macroscopic Brain Connectivity Using Multiple Modalities

**DOI:** 10.1155/2016/8128095

**Published:** 2016-01-24

**Authors:** Ni Shu, Yasser Iturria-Medina, Roberto C. Sotero

**Affiliations:** ^1^State Key Laboratory of Cognitive Neuroscience and Learning and IDG/McGovern Institute for Brain Research, Beijing Normal University, Beijing 100875, China; ^2^Center for Collaboration and Innovation in Brain and Learning Sciences, Beijing Normal University, Beijing 100875, China; ^3^McConnell Brain Imaging Center, Montreal Neurological Institute, McGill University, Montreal, QC, Canada H3A 2B4; ^4^Department of Radiology and Hotchkiss Brain Institute, University of Calgary, Calgary, AB, Canada T3A 2E1

With the advent of brain imaging techniques, the study of human brain connectivity* in vivo* becomes possible and has offered new insights into understanding the structural organization and work mechanisms of the human brain. Using different modalities of neuroimaging, both structural and functional brain connectivity can be mapped. The connectivity analysis has been widely applied into investigating the brain mechanisms of cognitive functions and neuropathology of different brain disorders. However, there are still some challenges when mapping, analyzing, and modeling the brain connectivity at different scales. This special issue aims at reflecting the advances in studies of brain connectivity, including both modeling methods and applications. In this special issue, different imaging techniques, such as structural MRI, diffusion MRI, and functional near-infrared spectroscopy (fNIRS), were employed in the studies of brain connectivity. The applications addressed hemispheric asymmetry, primary insomnia, and motor training. Also, the computational modeling methods and analysis toolbox of brain connectivity were introduced. The types of papers include review articles as well as original research.

For the modeling methods of brain connectivity, R. C. Sotero (2015) reviews computational models of phase-amplitude coupling (PAC) generation ranging from realistic networks of Hodgkin-Huxley neurons to neural mass models (NMMs) describing only the average activity of the neuronal populations involved and showed that NMMs are rich enough to provide a variety of PAC patterns. For the analysis toolbox of brain connectivity, J. Xu et al. (2015) developed a MATLAB software package that facilitates fNIRS-based human functional connectome data-analysis, which will be useful for the resting-state brain functional connectivity (FC) studies. For the application studies of brain connectivity, N. Shu et al. (2015) investigated the hemispheric asymmetry of brain white matter anatomical network which is constructed by diffusion MRI tractography and demonstrated that the topological asymmetries of the anatomical networks might reflect the functional lateralization of the human brain. L. Zhao et al. (2015) investigated the topological alterations of the structural covariance network for patient with primary insomnia, suggesting that insomnia might be related to underlying increase in brain network integration encompassing the sensory to motor networks, the default mode network, and the salience network and decrease in the integration between the sensory regions and the frontoparietal working memory network.

The special issue also contains several review papers: B. Wong et al. (2015) review structural and functional connectivity studies of bilingualism and examined different issues such as whether the language neural network is different for first- (dominant) versus second- (nondominant) language processing; the effects of bilinguals' executive functioning on the structure and function of the “universal” language neural network; the differential effects of bilingualism on phonological, lexical-semantic, and syntactic aspects of language processing on the brain; and the effects of age of acquisition and proficiency of the user's second language in the bilingual brain, and how these have implications for future research in neurolinguistics. T. D. Ben-Soussan et al. (2015) review the connectivity studies of quadrato motor training and proposed a general model tying cerebellar function to cognitive improvement, via neuronal synchronization as well as biochemical and anatomical changes.

In summary, the papers collected in this special issue cover a wide range of topics that are on the frontier of the methods and applications of brain connectivity.

